# Electrochromic Properties and Electrochemical Behavior of Marennine, a Bioactive Blue-Green Pigment Produced by the Marine Diatom *Haslea ostrearia*

**DOI:** 10.3390/md19040231

**Published:** 2021-04-19

**Authors:** Nellie Francezon, Mickaël Herbaut, Jean-François Bardeau, Charles Cougnon, William Bélanger, Réjean Tremblay, Boris Jacquette, Jens Dittmer, Jean-Bernard Pouvreau, Jean-Luc Mouget, Pamela Pasetto

**Affiliations:** 1 FR CNRS 3473 IUML, Mer-Molécules-Santé (MMS), Le Mans Université, Avenue Olivier Messiaen, CEDEX 9, 72085 Le Mans, France; Nellie.Francezon@univ-lemans.fr (N.F.); Mickael.Herbaut@Univ-lemans.fr (M.H.); Jean-Luc.Mouget@univ-lemans.fr (J.-L.M.); 2 Institut des Molécules et Matériaux du Mans, UMR CNRS 6283, Le Mans Université, Avenue Olivier Messiaen, CEDEX 9, 72085 Le Mans, France; Jean-Francois.Bardeau@univ-lemans.fr (J.-F.B.); Boris.Jacquette@univ-lemans.fr (B.J.); Jens.Dittmer@univ-lemans.fr (J.D.); 3 Laboratoire MOLTECH-Anjou UMR CNRS 6200 Faculté des Sciences, Université d’Angers, Bâtiment K, Boulevard Lavoisier, CEDEX, 49045 Angers, France; charles.cougnon@univ-angers.fr; 4 Institut des sciences de la mer de Rimouski, Université du Québec à Rimouski, 310 des Ursulines, Rimouski, QC G5L 3A1, Canada; william.belanger01@uqar.ca (W.B.); Rejean_Tremblay@uqar.ca (R.T.); 5 EA 1157, Laboratoire de Biologie et Pathologie Végétales (LBPV), Université de Nantes, F-44000 Nantes, France; Jean-Bernard.Pouvreau@univ-nantes.fr

**Keywords:** marennine, marennin, *Haslea ostrearia*, microalgae, diatom, natural blue pigment, cyclic voltammetry, redox properties, marine pigments, Raman spectrometry

## Abstract

Marennine has long been known as the unique peculiar pigment responsible for the natural greening of oysters. It is specifically produced by the marine diatom *Haslea ostrearia* and it is a natural blue molecule indeed promising for food industry because of the rarity of such non-toxic, blue-colored pigments. In the search for its still not defined molecular structure, investigation of the color changes with the redox state has been carried out combining different approaches. Reducing and oxidizing chemicals have been added to purified marennine solutions and a stable blue-green color has been confirmed for the oxidized state, while a yellow color corresponded to the reduced unstable state. Raman spectroscopy has been used to monitor changes in the Raman spectra corresponding to the different colored states, and cyclic voltammetry has allowed the detection of a redox system in which protons and electrons are exchanged. These findings show that marennine is a suitable stable blue pigment for use in food applications and help in the elucidation of the chromophore structure.

## 1. Introduction

Marennine has long been known as the unique peculiar pigment responsible for the natural greening of oysters [1], specifically produced by the marine diatom *Haslea ostrearia* (Gaillon) Simonsen (e.g., [2]). This microalga was first named *Vibrio ostrearius* [3], then renamed *Navicula ostrearia* by Bory [4] when he defined a new genus *Navicula* to encompass taxa for microorganisms that have a cell shape resembling a weaver’s shuttle, and finally *Haslea ostrearia* when Simonsen [5] proposed a new genus *Haslea* to include all pennate, lanceolate diatom species characterized by specific morphological features of their frustule, *H. ostrearia* being the generitype (see also Poulin et al., 2019 [6]). As to marennine, it is a water-soluble blue-green pigment, and its name is the usual form of marennin proposed by Lankester [7] in reference to the Bay of Marennes-Oléron (France), a region of the Atlantic west coast where oysters have been farmed for centuries. The Marennes-Oléron Bay is famous for the maturing and the fattening of oysters in special ponds called “claires à huîtres” where phytoplankton proliferates. In these ponds, from year to year but erratically, *H. ostrearia* becomes dominant, producing and releasing large amounts of marennine in seawater. Oysters feed on phytoplankton by filtering seawater and selecting particles in suspension. By doing so, marennine released in seawater is fixed on their gills that turn green [2], a phenomenon giving oysters an added-value due to their peculiar color and possibly flavor. Since inspiring works by Pouvreau et al., who demonstrated that marennine displays allelopathic [8] and antioxidant activities [9], other biological activities have been extensively studied in the last decade. Gastineau et al. [10] demonstrated that marennine has antibacterial, antiviral, and antiproliferative activities. Prasetiya et al. [11] confirmed its allelopathy against microalgae used for feed in aquaculture, but also evidenced some detrimental activity against marine invertebrates, although the effects are species- and age-specific [12]. Marennine can also be considered a toxic compound for reproduction and larval stages of different marine invertebrates, but not for adults [13,14]. Furthermore, significant strain- and species-dependent activities against *Vibrio* bacteria have been evidenced [15], with possible application in aquaculture as an anti-pathogen agent [16].

In the literature, marennine is usually treated as a pigment, possibly by analogy with other plant compounds like chlorophylls, carotenoids, flavonoids, or phycocyanin; however, it should be called a dye as it is naturally water soluble. Although the structure and biological function of marennine are hitherto not well known, it has potential applications in areas such as the food, cosmetic, textile, or even paper industries. Indeed, the use of natural pigments and dyes to replace synthetic ones is being more and more regarded, as the former are generally considered healthier as well as eco-friendly [17]. However, because the color blue is scarce in nature, only a few blue colorants are currently available. At present, only phycocyanin, anthocyanin, metal-chelates, and genipin-derived pigments are industrially used to produce blue hues [18]. For instance, phycocyanin produced by the cyanobacterium *Arthrospira* (*Spirulina*) *platensis* reached a global market size of $348 million in 2018, with a market estimation of $779 million by 2026 [19]. Therefore, marennine produced by *H. ostrearia* has received considerable attention for industrial and economic interests [20], as well as from researchers, with recent attempts to purify [21] and characterize [22] the molecule. If it has long been observed that the spectral characteristics of marennine change with pH, from purple-blue in an acidic medium to green in an alkaline one [2,22,23], less was known about the redox properties of the molecule, except remarkable antioxidant properties significantly higher than natural and synthetic antioxidants commonly used in food formulations [9]. The first part of the present work was inspired by preliminary experiments concerning the treatment of marennine solutions with reducing and oxidizing agents, which provoked the changes of color (J.B. Pouvreau, unpublished results). It was also shown that marennine was able to scavenge free radical oxygen species and reduce iron, but modifications of its structure were not described. The work herein presented is also linked to ongoing experiments on blue *Haslea* cells carried out to have a better understanding of the microalgae physiology. These results will help in clarifying the role of the pigment and the reasons why *Haslea* cells produce it, as there is still a debate about its intrinsic utility for self-defense because of the allelopathic [8,11], antiviral, and antibacterial properties [10,15], or for a “sunscreen” protection against solar radiation due to UV absorption.

Therefore, the objectives herein are to precise redox properties of marennine using different techniques, studying the impact of the extraction/purification methods on its chromophore, as well as monitoring its behavior in the presence of three common reductants used in food formulations. Thus, the present work is the first study of the electrochromic properties and electrochemical behavior of extracellular marennine produced by the diatom *H. ostrearia*.

## 2. Results and Discussion

### 2.1. Study of Marennine Color at Different pH

The color of marennine in aqueous solutions at different pH values was studied in the past on samples obtained using a semi-preparative procedure consisting in an ultrafiltration process followed by an anionic exchange chromatography [2,21]. In the present research work, the two types of samples used (small scale and scale-up production) were purified following different protocols; therefore, the study of the pH-depending spectrophotometric properties was repeated. A scheme describing the steps of the small-scale and scale-up production and the meaning of the acronyms used to name the fractions in this article is presented in Figure S1. 

**Figure 1:**
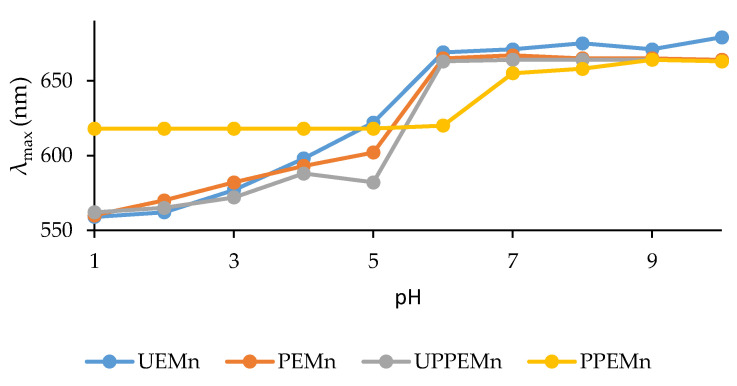
Evolution of maximum absorption wavelength (λ_max_) of marennine between 500 and 750 nm: maximum wavelength (nm) corresponding to pH values. Ultrafiltrated marennine (UEMn); Precipitated marennine (PEMn); Unretained Precipitated Purified marennine (UPPEMn); Purified Precipitated marennine (PPEMn).

The maximum absorption wavelengths of marennine (λ_max_) between 500 and 750 nm were determined as a function of pH (Figure 1). 

It was found that marennine λ_max_ varied after the different treatments during the purification processes. Ultrafiltrated marennine (UEMn) and precipitated marennine (PEMn) displayed the same behavior, with nearly superposed curves. This indicates that both extraction/concentration processes have similar effects on marennine chromophore. Color modification appeared after the retention of marennine in the purification column. Indeed, UPPEMn, which is immediately eluted from the column after the deposit, has a similar curve to UEMn and PEMn. Their color evolution in function of pH is comparable, from purple (λ_max_ 560 nm) to green (λ_max_ 670 nm) with a color switch between pH 5 and 6. As for marennine retained in the column and eluted with 50% of ethanol (PPEMn), one can observe a different behavior: purple color (560–580 nm) is lost for acidic pH, replaced by a bright blue (λ_max_ around 620 nm) and the color switch from blue to green occurs between pH 6 and 7. Marennine color is affected by organic solvents, so ethanol could be responsible for the blue color observed at acidic pH. However, purple color was never retrieved after total evaporation of ethanol from purified marennine samples and dilution with acidic water. It was also observed that an acidified marennine solution kept at room temperature for few days lost the purple color; if this solution was heated at 50 °C during 24–48 h, the loss of color occurred more quickly, as if the kinetic was accelerated. 

Interestingly, PPEMn blue color at acidic pH is very similar to intracellular marennine (IMn). Indeed, marennine contained in the algal cells (IMn) is blue at acidic pH, with a constant 620 nm maximum wavelength, whereas extracellular marennine (EMn) has a 570 nm maximum wavelength [22]. The change in the visible region of the absorption spectrum of marennine is known to occur during excretion of the pigment to the extracellular medium. However, this is the first time that a change backwards for the absorption in the visible region is observed. From this observation, we can only hypothesize that the chromophore of marennine might have recovered its original, intracellular form. 

The observed blue color stability (at 620 nm) of PPEMn from pH 1 to 6 is of great interest. Indeed, most of blue natural colorants lose their blue shades in acidic medium: anthocyanins turn purple-pink at low pH, natural blue anthraquinone or quinoid dyes become red or orange and phycocyanin from spirulina is unstable [24]. Purified marennine blue shade is unaffected by low pH, and remains unaltered even after acidic hydrolysis in harsh conditions (100 °C, 16 h, data not shown). Purified marennine could therefore be used as a blue colorant in acidic food and beverage products.

### 2.2. Marennine Redox Properties and Color Change

The dependence of the color of marennine on its oxidation state was at first investigated by adding different agents to the compound in solution. Experiments conducted by adding a sodium dithionite solution were appealing as it caused the yellowing of marennine. Sodium dithionite is a very versatile and powerful reducing agent, monitoring electron transfer reactions from various redox systems [25]. As dithionite reacts readily with oxygen (it quickly degrades into thiosulfate, sulfite, and elemental sulfur in aqueous solutions and, furthermore, it undergoes a rapid disproportionation in acidic solutions [26]) a first titration experiment was carefully carried out on UEMn after nitrogen bubbling and at pH 7. 

**Figure 2:**
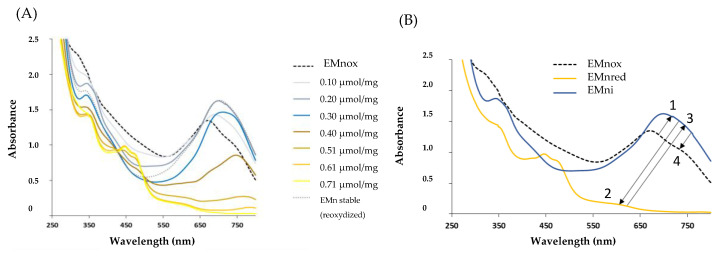
(**A**) Extracellular marennine (EMn) progressive reduction with increasing concentration of sodium dithionite and color evolution monitored by UV-visible spectrophotometry. (**B**) Marennine profiles sum up depending on oxidation degree: 1. shift from native oxidized EMnox to stable form EMni: first reduction; 2. shift from the stable to the reduced form EMnred: second reduction; 3. shift from reduced to stable form EMni: natural reoxidation with air oxygen; 4. shift from stable EMni to native EMnox form, forced reoxidation with hydrogen peroxide.

The initial absorption spectrum of UEMn at pH 7 displayed the maximum wavelength in the visible region at 670 nm (Figure 2A): with progressive addition of sodium dithionite (0.10 and 0.20 μmol/mg of UEMn), a bathochromic shift to the red wavelengths occurred (λ_max_ = 700 nm) and an increase in blue-green color. With greater amounts of dithionite, from 0.40 to 0.71 μmol/mg of UEMn, a rapid switch from blue to yellow color was visible and corresponded to a total decrease in absorbance in the 600 to 800 nm wavelength region, and a slight increase in the 400–500 nm wavelength region; therefore, a hypsochromic shift occurred (Figure 2A). The yellow reduced marennine was stable under nitrogen bubbling but, after having stopped bubbling, in contact with the oxygen of the air, UEMn spectrum came back to the spectrum of 0.20 μmol/mg of UEMn and not to the initial one. A slight bathochromic effect was observed. Reduction with sodium dithionite was reversible with oxygen and could be repeated at least 3 times. The initial spectrum of UEMn could be recovered by addition of the oxidizer hydrogen peroxide (150 μmol/mg). However, the reoxidation by the peroxide is a slow reaction so that an excess of oxidizer was added to observe a significant effect, which caused the dilution of the solution and the diminution of the absorbance (data not shown). Figure 2B sums up the different spectra of marennine during its reduction with sodium dithionite and reoxidation. 

**Figure 3:**
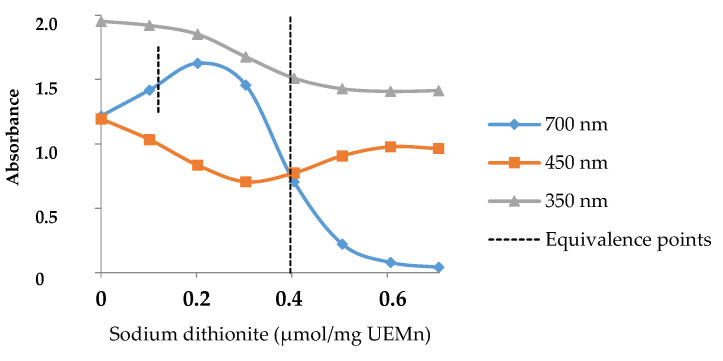
Color change of marennine with sodium dithionite addition and equivalence points.

To follow the evolution of marennine as a function of the addition of dithionite, the absorbance at three wavelengths was considered and presented in Figure 3.

The shoulders at 350 and 450 nm, which fluctuated, and the peak at 700 nm were chosen. Curves at 450 and 700 nm showed a first equivalent point at 0.12 µmol/mg of UEMn, which points out the transition from the initial EMn state to a more blue-green state. The second equivalent underlined by the three curves point at 0.40 µmol/mg indicated the clear shift from this state to the yellow form, and 0.61 µmol/mg of dithionite allowed a complete reduction of EMn. Therefore, three states of EMn can be described: a first initial or native state that corresponds to the oxidized form (EMnox), a second intermediate one (more blue, EMni), and a third reduced and yellow state (EMnred). Dithionite induced two reduction steps, which require—under the assumption of a molecular mass of 10 kDa [21]—less than 6 molecules of dithionite per EMn to obtain the yellow form, including 3 for the intermediate form. At least two redox couples are present: EMnox/EMni and EMni/EMnred. 

**Figure 4:**
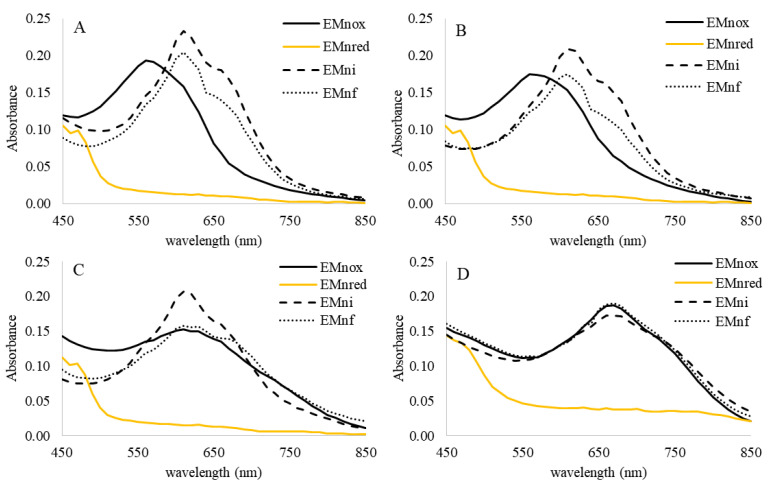
UEMn dithionite reduction and reoxidation. Visible spectra of UEMn (100 µL of UEMn at 1 g/L in phosphate buffer 0.1 M in plate of 96 wells) were measured at pH 2 (**A**), pH 3 (**B**), pH 4 (**C**), and pH 7.5 (**D**) for the native UEMn (EMnox), after addition of dithionite (20 µL, 10 mM) and further reoxidation (EMni). EMnf: control without dithionite addition after 24 h incubation in experimental conditions.

Given the differences observed on the spectrum as a function of pH, the behavior of EMn was also followed in more acidic pH. Spectrophotometric analysis in the range of pH 4 to 10 demonstrated the reversibility of the reduction with sodium dithionite (Figure 4). Since dithionite is not stable at acidic pH [26], the experiments were carried out by adding an excess of dithionite and followed in a 96-well plate. A control at pH 7.5 was maintained to compare the two methods. Despite some little color differences, UEMn, and PPEMn displayed the same behavior in presence of this reductant. Yellowing (EMnox) was observed at acidic pH (Figure S1 and Figure 4) but the reoxidation (EMni) was indeed markedly faster at acidic pH: 10, 12, and 15 min at pH 2, 3, and 4, respectively, against 20 min at pH 7.5. Unlike at pH 7.5, where the spectrum of the reoxidized form is close to the initial form (with an increase in absorbance at 600–800 nm and a slight bathochromic effect), at pH 2 and 3, a net shift of the λ_max_ is observed from 560 nm to 610 nm from EMnox to EMni, respectively, with an increase in absorbance (Figure 4A,B) and a color change from purple to blue. At pH 4, only the increase in absorbance is observed (λ_max_ remains at 610 nm; Figure 4C). At pH 7.5, under these conditions the spectra are similar (λ_max_ = 670 nm; Figure 4D). The pattern of the control, marennine without addition of dithionite (EMnf), was also followed. The spectra of EMnf at pH 4 and 7 were not modified (EMnf ≈ EMox), unlike at pH 2 and 3, whose λ_max_ moved as in the presence of dithionite from 560 to 610 nm (EMnf ≈ EMni) (Figure 4). The redox potential of EMnox/EMni is therefore dependent on the pH and the protonation state of the EMn. The EMnox/EMni in acid form (pH < pKa) seems to have a higher potential than the basic forms (pH 7.5), above the pKa previously determined at 4.74 [21]. EMnox at low pH is an oxidant in oxygenic conditions.

### 2.3. Marennine Reaction with Ascorbic Acid and Sodium Sulfite

**Figure 5:**
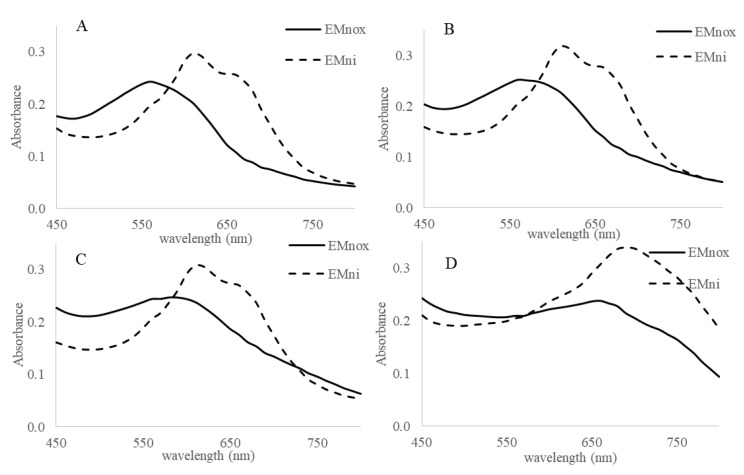
UEMn and ascorbate oxidoreduction. Visible spectra of UEMn (100 µL of UEMn at 1 g/L in phosphate buffer 0.1 M in plate of 96 wells) were measured at pH 2 (**A**), pH 3 (**B**), pH 4 (**C**) and pH 7.5 (**D**) for the native UEMn (EMnox), after addition of ascorbate (10 µL, 10 mM) (EMni).

Acid ascorbic, another reducer, was reacted with UEMn, (Figure 5). Under the different pH conditions, ascorbic acid induced a shift in the λ_max_ from 560 nm to 610 nm at pH 2 and 3; from 580 nm to 610 nm at pH 4, and from 660 nm to 690 nm at pH 7.5. An increase in absorbance was also observed, yellowing was however not observed. These data show that EMnox can be reduced to EMni by ascorbic acid and not to the fully reduced form, EMnred.

In the same conditions, ascorbic acid and sodium sulfite had no significant effect on PPEMn as no reductions or color changes were observed, even at high concentrations (Figures S3 and S4). Further, λ_max_ of PPEMn remains stable at 620 nm below pH 6 (Figure 1). PPEMn seems to have properties close to EMni. As sodium sulfite and ascorbic acid are two very common preservatives in food industries, their lack of influence on marennine color is of interest for a future use of marennine as a stable blue colorant in processed food.

**Figure 6:**
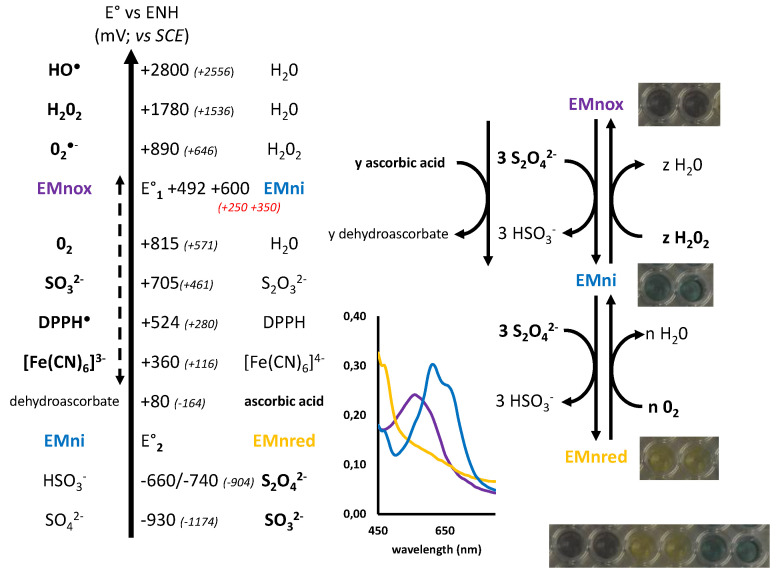
Estimation of standard redox potentials of marennine couples and corresponding colors.

The comparison of the standard redox potential (E° vs. ENH, 25 °C) of the various reducing agents and the reactions observed makes it possible to propose a first redox potential (E°_1_) of the EMnox/EMni couple between +1780 mV (H_2_O_2_/H_2_O) and +815 mV (O_2_/H_2_O) for pH below pKa, and between +815 mV (O_2_/H_2_O) and + 80 mV (dehydroascorbate/ascorbic acid) for pH above pKa. Indeed, the EMni state is not reoxidized in the presence of oxygen but with oxygen peroxide and at pH below pKa, EMnox is reduced to EMni. EMnox and EMni are reduced by dithionite (HSO_3_^−^/S_2_O_4_^2−^; E° = −660/−740 mV) but yellowing is not observed by addition of ascorbic acid (dehydroascorbate / ascorbic acid; E° = + 80 mV). Therefore, E°_1_ is above +80 mV. Moreover, a second redox potential (E°_2_) of EMni/EMnred must be between +80 and −660/740 mV. These data are summarized in Figure 6. The E°_1_ data were determined by electrochemical experiments (see Section 2.5). 

The UV-visible profile of marennine reduction with sodium dithionite is very similar to the iron phthalocyanine one, a metal complex [27], with a rapid first reduction from green to blue and a second, slower, from blue to brown. However, the absence of transition metals in the elemental analysis of purified marennine [22] dismissed this hypothesis. Reversible yellowing or bleaching of several natural blue molecules with sodium dithionite were reported in the literature, with different mechanisms, such as indigo dye [28], methyl viologen [29], or phycocyanin [30]. One of the hypotheses about marennine chromophore was that it could be a substituted anthraquinone. Studies about anthraquinones derivatives from marine-derived fungi showed that there is a connection between structural diversity and biological activities [31], and looking at the colors and structures of these compounds may suggest that in marennine chromophore, two different quinone systems exist and are reduced at different potentials. The absorption in the UV region also goes along this line, with a decrease in the intensity of the peak at 320 nm (quinoid band) after the first reduction, and the appearance (or increase) of the absorbance of the peak at 350 nm that could be associated with new benzenoid transitions.

These redox potentials and the associated changes in UV-visible spectra raise questions related to the activities and biological functions of marennine and about the genetic history that brought the microalgae to the production of the pigment.

### 2.4. Raman Spectroscopy

**Figure 7:**
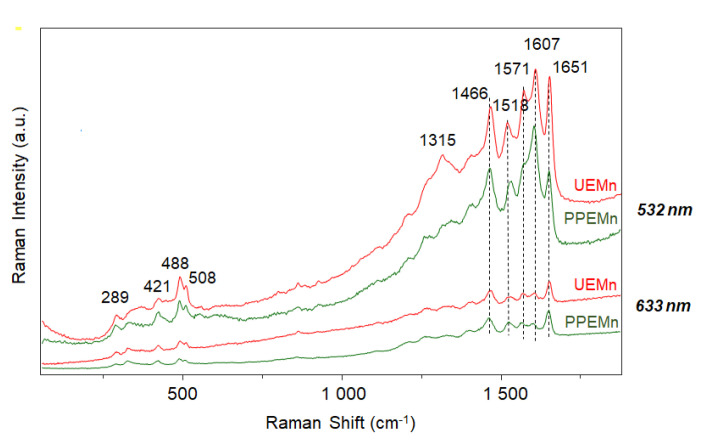
Raman spectra of marennine (UEMn, PPEMn) recorded with an incident wavelength at 532 and 633 nm.

Micro-Raman spectroscopy is a powerful non-destructive technique for revealing differences between uncommon biomolecules. Compared to other spectroscopic techniques, Raman spectroscopy needs a small amount of matter and limited sample preparation and offers a high spectral resolution and narrow spectral bands. In the case of colored compounds, the Resonance Raman (RR) spectroscopy can help to reveal an intensity enhanced spectrum from a chromophore without observing any contribution from a complex biological system [10,20,32,33]. By adjusting the incident wavelength of the laser illumination, it is possible to both reveal peaks in the Raman spectrum related to specific vibrational modes of functional groups (even at low concentration) and limit the intrinsic fluorescence background. The highest signal-to-noise spectra of marennine (Figure 7) were recorded using a green excitation (532 nm, Nd: YAG laser). The Raman spectra of UEMn and PPEMn are similar with well-resolved bands between 1200 and 1700 cm^−1^ and between 200 and 600 cm^−1^. The interpretation of RR spectrum is usually not trivial since the intensity of the peaks and the peak positions of the chromophores are characteristic of both vibrational and electronic properties. Nevertheless, it is admitted that the marker bands can provide an interesting insight into the electronic behavior of chromophore when environmental conditions are modified. 

In a first attempt, Raman comparison of UEMn and PPEMn confirmed that both purification methods led to similar purified chromophores, with comparable distinctive bands (Figure 7). Small variations in intensity could be observed for the peaks located at 1651 and 1571 cm^−1^ with lower intensities in the case of PPEMn. Such Raman behavior can result from changes of partial charges in the proximity of phenyl rings [10] or quinone segments [34,35].

A Raman shift of 11 cm^−1^ is also observed for the band located at 1518 cm^−1^ for UEMn. Both of these modifications are likely due to the introduction of a larger amount of trifluoroacetic acid in the marennine solution for PPEMn (0.5%, *v/v*) compared to UEMn (0.1%, *v/v*), confirming that the marennine chromophore is sensitive to pH. 

**Figure 8:**
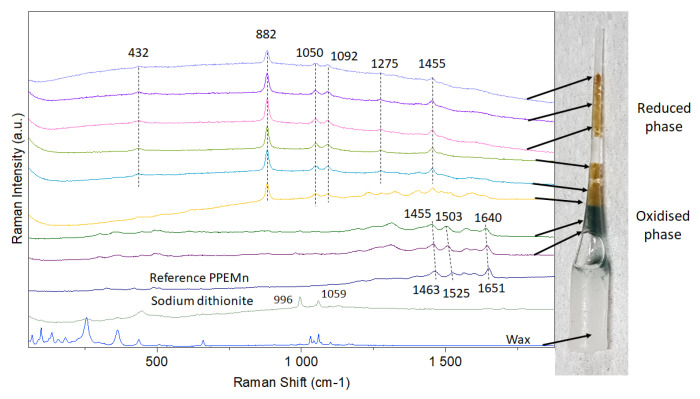
Raman spectra (recorded at 633 nm) of the reduced (yellow-brown) and oxidized phases (green) contained in the sealed capillary. The spectra of wax, sodium dithionite powder, and marennine PPEMn are presented for comparison.

As previously shown, the introduction of sodium dithionite into the marennine solution can cause a yellowing of the solution, likely due to the formation of a reduced state of the chromophore. Consequently, it was decided to make a homemade sealed glass cell in order to stabilize the reduced phase long enough to collect the Raman vibrational bands associated with this electronic state. Several attempts were necessary to successfully change the color of the marennine powder without going into a liquid form, which resulted systematically in a strong increase of the fluorescence background. In Figure 8, the homemade glass cell is presented and, for the first time, the spectrum of the marennine in its reduced phase; the main well-resolved characteristic bands are observed at 432, 882, 1050, 1092, 1275, and 1455 cm^−1^. These results clearly show that the appearance of these new Raman peaks is associated with the color change and, interestingly, the strong peaks located at 1651, 1607, and 1571 cm^−1^ in PPEMn were no longer visible in the reduced form of marennine, probably due to the strong fluorescence baseline. 

The modification of the Raman spectra with the introduction of sodium dithionite allows us to hypothesize that the change in the state of the chromophore may come from a reduction of semiquinones and/or benzoquinones to hydroquinones. Indeed, the Raman spectrum of reduced state is similar to simulated and experimental data recently reported by Cabrera-Alonso et al. [36]. Based on previous work [37], a tentative attribution of the main vibrational bands can be made. The strongest band at 882 cm^−1^ can be assigned to the C-C stretching mode, the band at 432 cm^−1^ to the C-C-C planar deformation vibration mode, and the band at 1275 cm^−1^ to the C-O stretching mode. 

After several hours, a change in the color of the marennine was observed at the edge of the capillary due to a reoxidation of the reduced form, and capillaries were assumed at first not being airtight. It was later found that the change in color was caused by the progressive degradation of dithionite in water; however, this gave the opportunity to study the phase transition from the reduced to the reoxidized state (green marennine). In Figure 8, it can also be noticed that the vibrational bands associated with the reoxidized state are shifted in comparison with those of native marennine (noted as reference PPEMn). Indeed, the more intense bands observed at 1463, 1525, and 1651 cm^−1^ for the native marennine PPEMn are significantly shifted towards 1455, 1503, and 1640 cm^−1^, respectively. This feature can likely be explained by the uncompleted oxidation process. These results are consistent with the spectrophotometric observations and the possibility of reversibly modifying the electronic properties of the chromophore by introducing reducing and oxidizing agents. Moreover, even if at this stage Raman information is insufficient to propose a complete molecular structure of the chromophore, it can be supposed that phenyl rings and quinone systems might be associated with the blue color. This hypothesis is also supported with data from nuclear magnetic resonance (NMR) spectra, which evidenced aromatic protons in the isolated blue fractions [20]. It must be underlined that this is the first time that Raman spectra are recorded for different unstable electronic states associated with color change (for native (blue-green), reduced (yellow-brown), and reoxidized (green) phases). These results will be further used as a reference to compare the behaviors of marennine-like pigments produced by other blue *Haslea* species [38,39,40], according to reduced or oxidized states with marennine’s one (ongoing work). 

### 2.5. Electrochemical Behavior 

**Figure 9:**
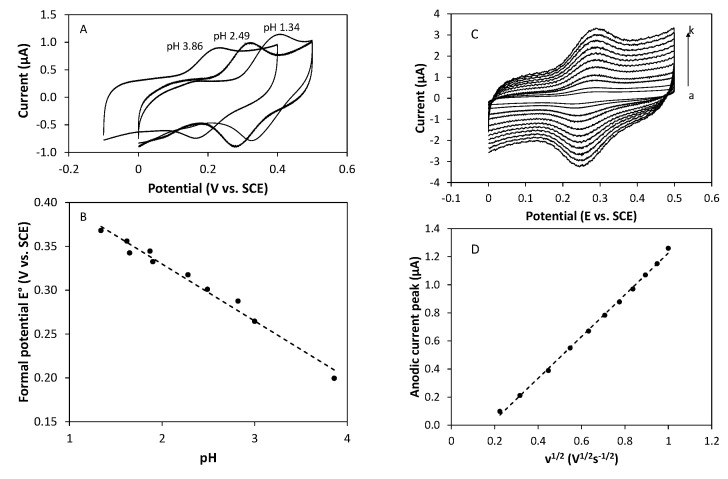
(**A**) Cyclic voltammograms recorded at 100 mV s^−1^ on a glassy carbon electrode in phosphate buffer marennine solutions (1 mg mL^−1^) at different pH. (**B**) Plot of the formal potential vs. solution pH. (**C**) Cyclic voltammograms recorded on a glassy carbon electrode in a phosphate buffer solution (pH 3) containing marennine (1 mg mL^−1^) at different scan rates: (a) 50 mV s^−1^, (b) 100 mV s^−1^, (c) 200 mV s^−1^, (d) 300 mV s^−1^, (e) 400 mV s^−1^, (f) 500 mV s^−1^, (g) 600 mV s^−1^, (h) 700 mV s^−1^, (i) 800 mV s^−1^, (j) 900 mV s^−1^, and (k) 1000 mV s^−1^. (**D**) Plot of the anodic current peak intensity vs. the square root of the scan rate.

In parallel to the redox experiments effectuated by adding chemicals to the marennine solution, cyclic voltammetry studies were conducted. The behavior of marennine aqueous solutions at different pH values was recorded varying the potential. Figure 9A) displays typical cyclic voltammograms (CVs) of marennine (C = 1 mg·mL^−1^) in phosphate buffer solution in a range of pH comprised between 4 and 1. At each pH value, a reversible pair of peaks is visible, as expected for a rapid one-electron transfer. The ratio of cathodic to anodic peak current is close to unity for all the pH values investigated, demonstrating that the oxidation product of marennine is stable in the time scale of the experiment. The shift of the formal potential of about 60 mV per pH unit agrees with a Nernstian behavior predicted for a coupled electron-proton transfer, implying so many electrons as protons (Figure 9B). Intriguingly, above pH 6, marennine remains electrochemically silent. These data agree with the variation of the first redox potential (E°1, EMnox/EMni) observed as a function of the pH (Figure 6) and the second redox potential (E°2, EMni/EMnred) could not be observed with this method (potential range −0.5–+0.6 V vs. ECS or −0.25–+0.75 V vs. ENH). The lack of response above pH 6 may also underline that the first redox potential is only valid for the acid form and absent from the basic form. 

CVs of marennine recorded at different scan rates with ohmic drop compensation are presented in Figure 9C. Over all the range of scan rate experienced, CVs exhibit reversible behavior with a peak-to-peak potential difference of around 60 mV and a current peak intensity increasing linearly with the square root of the scan rate (Figure 9D), which is in agreement with the predicted value for a mass transfer-controlled one-electron reversible exchange [41].

In this case, the diffusion coefficient of marennine can be calculated from the slope of the dependence of the anodic peak current *i_p_* on the square root of the scan rate, according to the Randles–Sevcik equation:

(1) $$i_{p}=0.446\mathrm{nFA}{\left(\frac{\mathrm{nF}}{\mathrm{RT}}\right)}^{1/2}{\mathrm{CD}}^{1/2}v^{1/2}$$

where n is the number of electrons transferred, *A* is the electrode area, D is the diffusion coefficient, and *ν* is the scan rate (F, R, T, and C have their usual meanings). The main difficulty here is that both the molecular weight and the number of electrons exchanged are unknown, since the chemical structure of marennine remains unclear. Assuming that the reversible system previously obtained corresponds to a one-electron exchange, as it agrees well with the peak-to-peak potential separation in the CVs, a complete electrolysis was achieved onto a carbon plate as working electrode to approximate the molecular weight. It was observed that an anodic charge of 19 mC must be consumed for the anodic current down to zero, which corresponds to a molecular mass equal to around 10,150 g mol^−1^. Remarkably, this value is in very good agreement with other published estimations of the molecular weight obtained by migration on acrylamide gel electrophoresis (10,000–12,000 Da) or mass spectrometry, which gives a value of 10,751 Da for the pure intracellular marennine and 9893 Da for the pure extracellular marennine [22]. It was noted that the good agreement between these different techniques reinforces the conclusion that the reversible system obtained by cyclic voltammetry corresponds to a one-electron-one proton transfer, depending on the pH.

The diffusion coefficient thus determined was found to be equal to 6.2 × 10^−11^ m^2^.s^−1^, which corresponds well to the value determined by diffusion-ordered two-dimensional NMR spectroscopy (D ≈ 1 × 10^−10^ m^2^ s^−1^ in D_2_O) [20].

A good account of the diffusion process is given by the Stokes-Einstein equation:

(2) $$D_{\mathrm{SE}}=\frac{k_{B}T}{6\pi \eta R}$$

where k_B_ is the Boltzmann constant, T is the absolute temperature, η is the viscosity of the solvent, and R represents the hydrodynamic radius of the tracer envisioned as a spherical particle. Anticipating a good correlation between experimental and theoretical values of the diffusion coefficient, the molecular size of marennine can be approximated by the Stoke-Einstein equation. The molecular volume of marennine envisioned as a spherical shaped molecule was approximated to 245 nm^3^.

## 3. Materials and Methods

### 3.1. Algal Production and Marennine Purification

Extracellular Marennine (EMn) was obtained using two purification procedures associated with two different size cultures: in small-scale marennine production (20 L), the EMn in the supernatant was purified by precipitation using chemicals (suitable for lab scale batches, quick), and in scale-up production (100 L) by mechanical purification using ultrafiltration (for scaled-up batches, longer).

A small production batch was carried out with a non-axenic *Haslea ostrearia* strain NCC (Nantes Culture Collection) 495, cultured at 16 ± 1 °C in a temperature-controlled room, at an irradiance of 100 µmol m^−2^ s^−1^ provided by Philips TLD 36 W/965 fluorescent tubes (14 h/10 h, light/dark cycle). Cultures were grown in 500 mL Erlenmeyer flasks containing 250 mL of an autoclaved artificial seawater, prepared from a commercial sea salt mix (Instant Ocean, Aquarium Systems^®^, pH 7.6 ± 0.2, salinity 32 ppm, see Falaise et al. [13]), with an enrichment solution as described in Mouget et al. [42]. The culture medium (20 L) was filtered on 15 μm and 1.4 μm cut-off paper filters (Grosseron, Coueron, France) to remove cell residues. Then, a specific precipitation method using acid and base was carried out according to the procedure described in the patent n° (FR2019/052933) to concentrate the filtered supernatant: a 10 M NaOH aqueous solution was added dropwise until the complete discoloration of the supernatant. The blue precipitate formed was collected by centrifugation (4000 rpm, for 5 min) and re-dissolved with the minimum amount of formic acid. This blue concentrated extract (PEMn: Precipitated Extracellular Marennine) was dialyzed against ultrapure water for 48 h (MWCO 1 kDa, Spectrum). After dialysis, the deep blue retentate was further purified using a solid phase extraction 20 g C-18 cartridge (Fischer Scientific, Illkirch, France), using a water-ethanol gradient from 1:0 to 0:1 ratio, acidified with a 0.5% (*v/v*) TFA aqueous solution (trifluoroacetic acid, Aldrich Merck KGaA, Darmstadt, Germany). The marennine interacting with the C-18 cartridge was recovered with a water-ethanol 1:1 mixture, although a minority of marennine was not retained and was directly eluted with water (UPPEMn: Unretained Purified Precipitated Extracellular Marennine). The water-ethanol 1:1 mixture was then evaporated in order to recover marennine as a dry powder (PPEMn: Purified Precipitated Extracellular Marennine).

Scale-up marennine production was obtained from the strain *Haslea ostrearia* NCC 136 isolated from Bourgneuf Bay, France. The procedure of strains up-scale culturing, marennine extraction by ultrafiltration process and storage of culture medium (blue water) followed the procedure described by Turcotte et al. [16]. In brief, 100 L of photobioreactor was used and extracellular marennine was extracted through a two-step ultrafiltration process (30 and 3 kDa) with cartridges fitted with regenerated cellulose spiral membranes (Prep/Scale Spiral Wound TFF-6 0.54 m2 Emd Millipore). The concentration of ultrafiltrated marennine (UEMn: Ultrafiltrated Extracellular Marennine) was determined by spectrophotometry at 656 nm with the specific extinction coefficient of 12 L g^−1^ cm^−1^ (Pouvreau et al. [43]). For Raman spectroscopy, UEMn was further purified on SPE C-18 as previously detailed, using TFA 0.1% (*v/v*).

### 3.2. Redox Experiments in Solution and Color Change

The color evolution of UEMn, PEMn, UPPEMn, and PPEMn solutions at different pH was assessed by spectrophotometry. Each sample was diluted (1 mg/mL) with phosphate buffers at 100 mM, in the pH range 1–10. Three reductants were tested (100 mM aqueous solutions extemporaneously): sodium dithionite, sodium sulfite, and ascorbic acid. Spectrophotometric measurements were realized in a 96-well microplate reader (Bioteck Instruments Inc., Winooski, VT, USA) and in a 2 mL quartz cuvette closed with a septum for oxygen-free experiments, using UV-visible spectrophotometers UV-3100PC (VWR, Leuven, Belgium) and a Varian UV-Visible spectrophotometer, model Cary 100. Freshly prepared, nitrogen-purged reagents were injected through the septum into the samples using a microliter glass syringe Hamilton 50 µL (Hamilton, Giarmata, Romania). The samples were titrated until fully reduced (yellow), then briefly exposed to air and shaken to induce reoxidation. In a second step, oxidization was forced using hydrogen peroxide 35 wt % (Aldrich, Merck KGaA, Darmstadt, Germany).

### 3.3. Resonance Raman Spectroscopy

Raman spectra of UEMn and PPEMn were recorded at room temperature using a DXR Raman microscope (Thermo Fisher Scientific, Inc., Waltham, MA, USA) equipped with a 1800 lines/mm diffracting grating. The use of a glass capillary allowed the recording of well-resolved Raman spectra of marennine with a reduced intrinsic fluorescence baseline (Figure S5). The capillary was first soaked into the solution of interest and after drying, the glass was covered with a thin film. A Mplan 10× magnification objective was used to focus the laser onto the glass capillary surface and collect the scattered light in a backscattering geometry. Raman measurements were carried out at low laser power (<1 mW at the sample surface) to avoid unintentional heating alteration of the organic molecules. The highest quality spectra (highest signal-to-noise) of marennine were obtained over a wavenumber range of 55–1875 cm^−1^ using green excitation (532 nm, Nd: YAG laser).

The optimization of the experimental Raman protocols and the realization of a homemade sealed glass cell made it possible to record for the first time a Raman spectrum of the reduced phase (by means of the use of dithionite solution). Raman spectra were recorded twice (for averaging) over a wavenumber range of 55–1875 cm^−1^ using the 633 nm (HeNe) excitation line.

The Raman spectra were systematically shifted in the figures (without any baseline correction) for clarity.

### 3.4. Cyclic Voltammetry

Electrochemical measurements of EMn were made in 0.1 M of phosphate buffer solutions adjusted to a pH comprised between 4 and 1 using HCl. KCl 0.1 M was added to the phosphate buffer solutions to maintain the ionic strength constant. A 3-electrode cell configuration was used. The working electrode was a glassy carbon electrode from Bioanalytical Systems Inc. (West Lafayette, IN, USA, model MF-2012; 3 mm in diameter) and the counter electrode was a platinum wire. All potential values were referred to the SCE system. A bipotentiostat from CH Instruments (model 920C) was used.

## 4. Conclusions

Recent research on marennine chemical structure has led to the investigation of its electrochemical behavior. In this article, observations about the pigment color changes with pH, addition of reducing and oxidizing agents, and potential were gathered using complementary techniques. Marennine’s chromophore demonstrated to be unaffected by acidic and basic treatments used for its isolation. Indeed, it has been shown that either chemical or mechanical purification is suitable for marennine recovery in a quite purified form.

Marennine’s color has been demonstrated to be relatively stable for food application. Its blue-green shade, associated with the stable state, remains constant in the presence of common food antioxidants and preservatives such as ascorbic acid and sodium sulfite. Therefore, marennine appears to be a good candidate as a new food colorant, especially in acidic preparations.

The set-up of an original and efficient protocol to work in a controlled environment to stabilize the redox phases of marennine allowed the recording of the Raman spectrum associated with reduced and oxidized phases. Cyclic voltammetry experiments performed on marennine allowed us to illustrate the electrochemical behavior of the molecule and its electroactivity at acidic pH, thus providing the first evidence of a reversible redox system in which protons and electrons are exchanged.

## Supplementary Materials

The following are available online at https://www.mdpi.com/article/10.3390/md19040231/s1. Figure S1. Scheme representing the two procedures used to purify extracellular marennine (EMn) from the supernatant of the microalgae culture medium. Figure S2. Monitoring of sodium dithionite reduction absorption spectra of marennine, UEMn, and PPEMn at different pH values (2.5, 4, 6, 7.4, and 10). Green arrows represent reductions and red arrows reoxidations. (A) Reduction profiles of marennine with 10 mM of sodium dithionite; (B) Reduction profiles of marennine with 20 mM of sodium dithionite; (C) Reoxidation profile of reduced marennine, exposed to the ambient air. Figure S3. Marennine PPEMn reduction with sodium sulfite, monitored by spectrophotometry. The amount of sodium suphite reacting is expressed in μmol per mg of marennine. Figure S4. Marennine PPEMn reduction with ascorbic acid, monitored by spectrophotometry. The amount of ascorbic acid reacting is expressed in μmol per mg of marennine. Figure S5: Raman spectra of marennine (UEMn) recorded on a film covering a glass capillary. Λ = 532 nm.
