# Supplementary material for: Electrochromic Properties and Electrochemical Behavior of Marennine, a Bioactive Blue-Green Pigment Produced by the Marine Diatom Haslea ostrearia
Source: Mar Drugs. 2021 Apr 19;19(4):231. doi: 10.3390/md19040231 (PMC8073169; doi:10.3390/md19040231)
Supplement: Supplementary file 1 [file marinedrugs-19-00231-s001.zip › marinedrugs-1159328-supplementary(1).pdf]

Supporting Information

Electrochromic Properties and Electrochemical Behavior of Marennine, a Bioactive Blue-Green Pigment Produced by the Marine Diatom *Haslea ostrearia*

Nellie Francezon [1,2], Mickaël Herbaut [1,2], Jean-François Bardeau [2], Charles Cougnon [3], William Bélanger [4], Réjean Tremblay [4], Boris Jacquette [2], Jens Dittmer [2], Jean-Bernard Pouvreau [5], Jean-Luc Mouget [1] and Pamela Pasetto [2,*]

1. FR CNRS 3473 IUML, Mer-Molécules-Santé (MMS), Le Mans Université, Avenue Olivier Messiaen, CEDEX 9, 72085 Le Mans, France; Nellie.Francezon@univ-lemans.fr (N.F.); Mickael.Herbaut@Univ-lemans.fr (M.H.); Jean-Luc.Mouget@univ-lemans.fr (J.-L.M.)
2. Institut des Molécules et Matériaux du Mans, UMR CNRS 6283, Le Mans Université, Avenue Olivier Messiaen, CEDEX 9, 72085 Le Mans, France; Jean-Francois.Bardeau@univ-lemans.fr (J.-F.B.); Boris.Jacquette@univ-lemans.fr (B.J.); Jens.Dittmer@univ-lemans.fr (J.D.)
3. Laboratoire MOLTECH-Anjou UMR CNRS 6200 Faculté des Sciences, Université d'Angers, Bâtiment K, Boulevard Lavoisier, CEDEX, 49045 Angers, France; charles.cougnon@univ-angers.fr
4. Institut des sciences de la mer de Rimouski, Université du Québec à Rimouski, 310 des Ursulines, Rimouski, QC G5L 3A1, Canada; william.belanger01@uqar.ca (W.B.); Rejean_Tremblay@uqar.ca (R.T.)
5. EA 1157, Laboratoire de Biologie et Pathologie Végétales (LBPV), Université de Nantes, F-44000 Nantes, France; Jean-Bernard.Pouvreau@univ-nantes.fr (J.-B.P.)

* Correspondence: pamela.pasetto@univ-lemans.fr

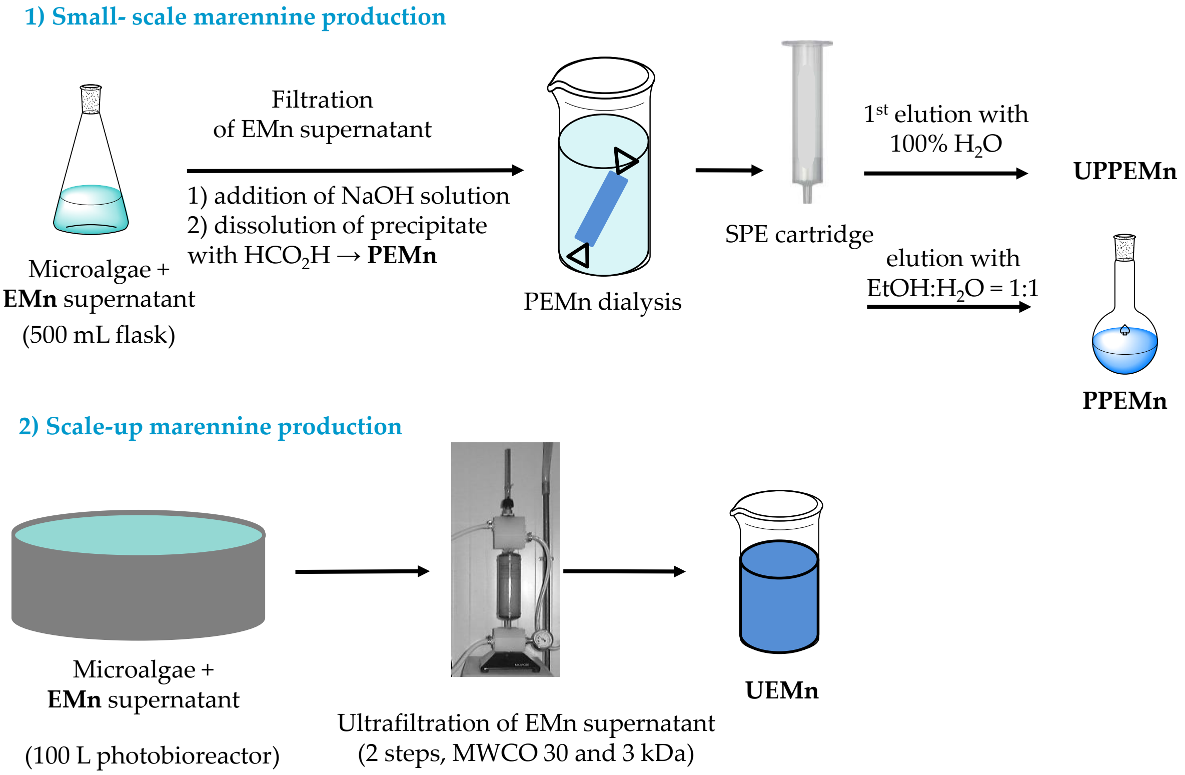

Figure S1. Scheme representing the two procedures used to purify extracellular marennine (EMn) from the supernatant of the microalgae culture medium.

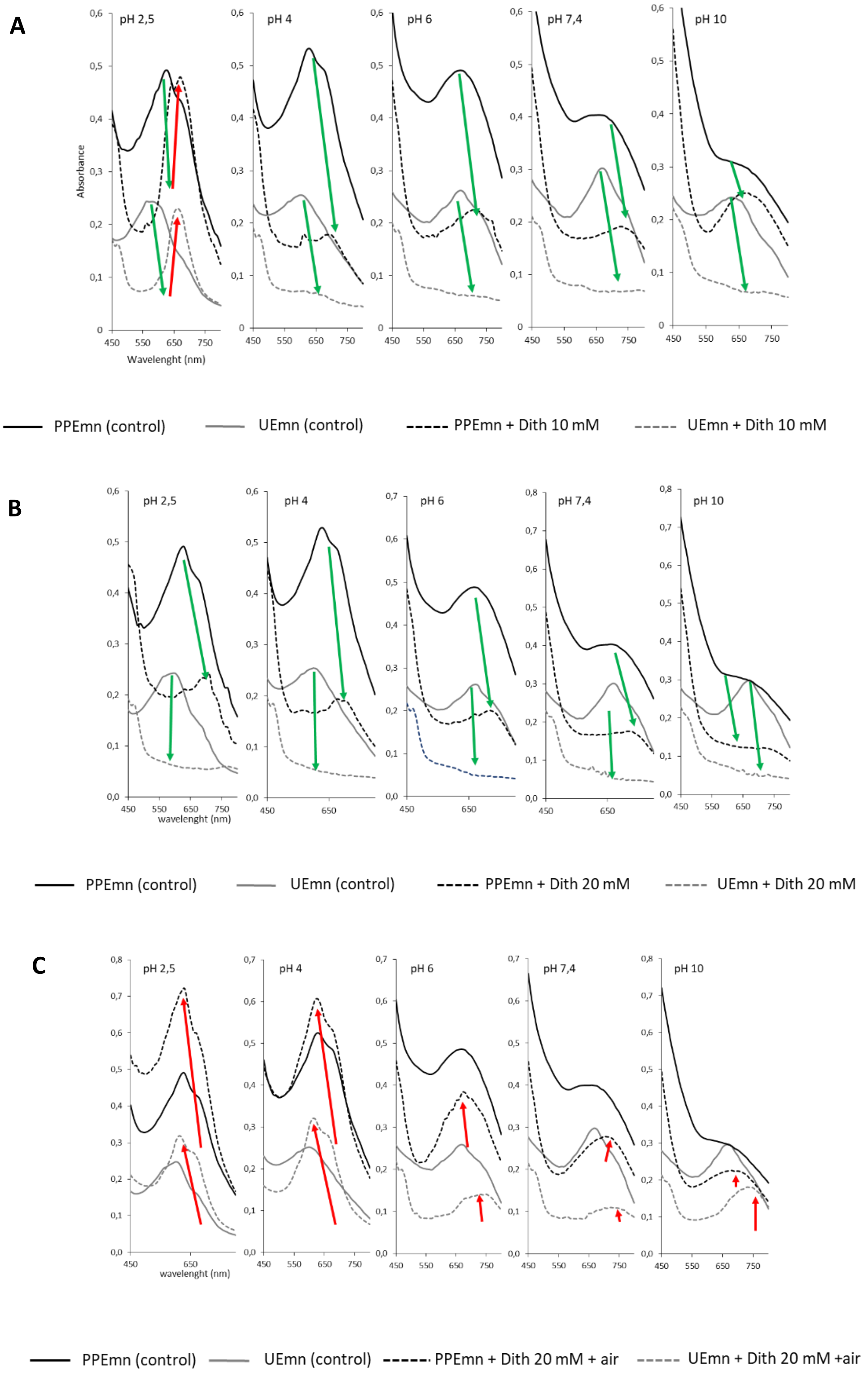

Figure S2. Monitoring of sodium dithionite reduction absorption spectra of marennine UEMn and PPEMn at different pH values (2.5, 4, 6, 7.4 and 10). Green arrows represent reductions and red arrows reoxidations. (A) Reduction profiles of marennine with 10 mM sodium dithionite; (B) Reduction profiles of marennine with 20 mM sodium dithionite; (C) Reoxidation profile of reduced marennine, exposed to the ambient air.

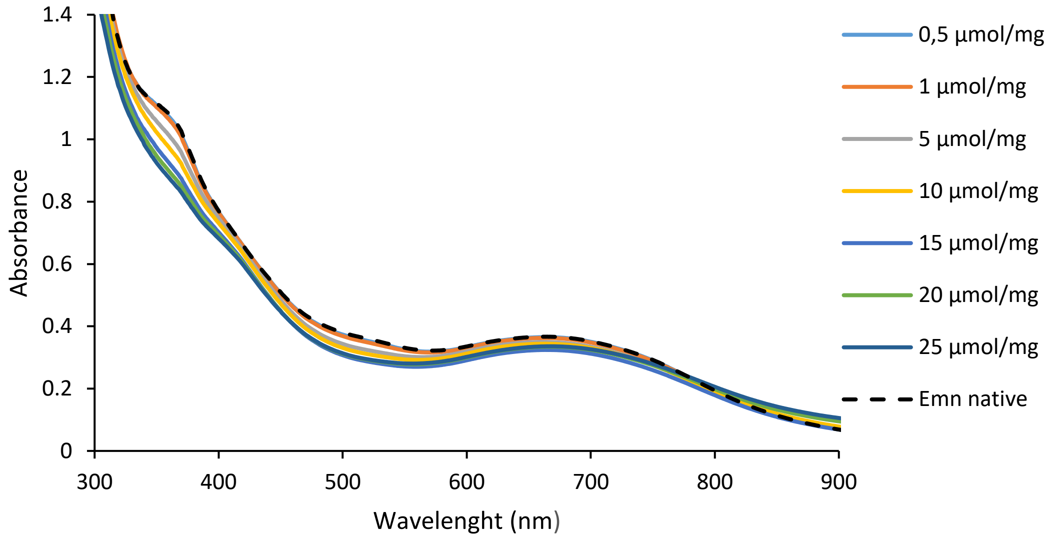

Figure S3. Marennine PPEMn reduction with sodium sulfite, monitored by spectrophotometry. The amount of sodium sufite reacting is expressed in µmol per mg of marennine.

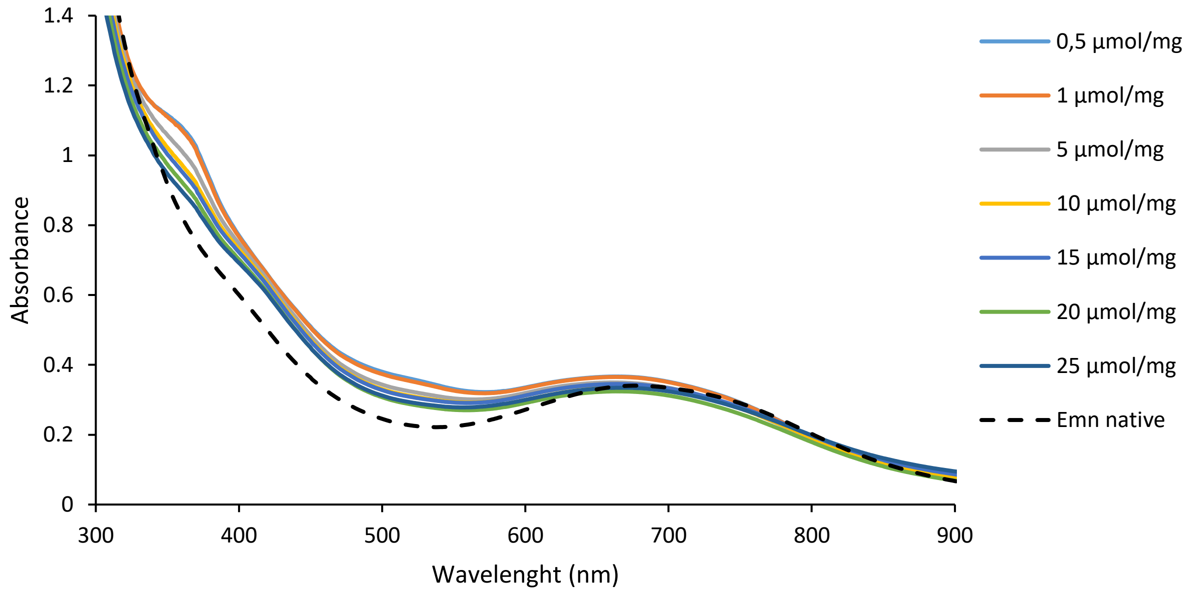

Figure S4. Marennine PPEMn reduction with ascorbic acid, monitored by spectrophotometry. The amount of ascorbic acid reacting is expressed in µmol per mg of marennine.

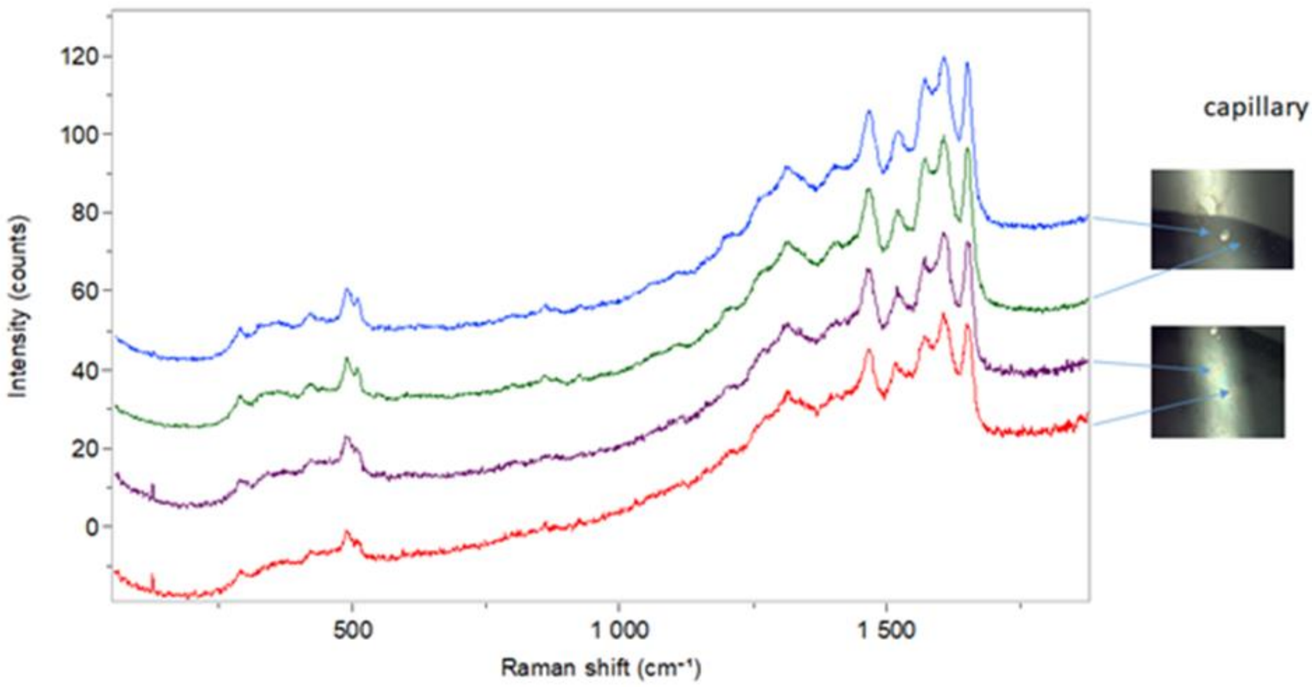

Figure S5: Raman spectra of marennine (UEMn) recorded on a film covering a glass capillary. λ=532 nm
